# Living with severe perinatal depression: a qualitative study of the experiences of labour migrant and refugee women on the Thai-Myanmar border

**DOI:** 10.1186/s12888-018-1815-7

**Published:** 2018-07-16

**Authors:** Gracia Fellmeth, Emma H. Plugge, Suphak Nosten, May May Oo, Mina Fazel, Prakaykaew Charunwatthana, François Nosten, Raymond Fitzpatrick, Rose McGready

**Affiliations:** 1 Nuffield Department of Population Health, University of Oxford, Old Road Campus, Oxford, OX3 7LF UK; 2 Shoklo Malaria Research Unit, Mahidol-Oxford Tropical Medicine Research Unit, Faculty of Tropical Medicine, Mahidol University, Mae Sot, Tak Province, 63110 Thailand; 3 Centre for Tropical Medicine and Global Health, Nuffield Department of Medicine, University of Oxford Peter Medawar Building for Pathogen Research, South Parks Road, Oxford, OX2 UK; 4 Department of Psychiatry, University of Oxford, Warneford Hospital, Warneford Lane, Oxford, OX3 7JX UK; 5 Faculty of Tropical Medicine, Mahidol University, 420/6 Ratchawithi Road, Ratchathewi, Bangkok, 10400 Thailand

**Keywords:** Perinatal depression, migrant, refugee, qualitative, pregnancy, post-partum, low- and middle-income

## Abstract

**Background:**

Perinatal depression is an important contributor to maternal morbidity and mortality worldwide. Migrant women, particularly those resettling within low- and middle-income settings, are at increased risk of perinatal depression due to multiple stressors experienced before, during and after migration. Evidence on migrant perinatal mental health to date has focused largely on women in high-income destination countries, leaving the voices of displaced women in low-income settings unheard. This study addresses the current evidence gap by exploring the experiences of migrant women living on the Thai-Myanmar border.

**Methods:**

In-depth interviews were conducted with pregnant and post-partum labour migrant and refugee women on the Thai-Myanmar border who had been diagnosed with severe depression. An interview guide covering women’s current and past life experiences, social support and the impact of depression on social and occupational functioning was used as a prompt. Thematic analysis was used to identify themes emerging from women’s narratives.

**Results:**

Eleven pregnant and post-partum women with severe perinatal depression took part. Participating women provided extensive insight into the many difficult aspects of their lives that they perceived as contributing to their depression status. Predominant themes emerging from women’s narratives included difficult relationships with partners, challenging life situations, mechanisms for coping with depression and impressions of mental health care.

**Conclusions:**

Labour migrant and refugee women with severe perinatal depression face a wide range of chronic stressors at the individual, household and community levels that are likely to have both short- and long-term negative effects on their mental well-being and day-to-day functioning. Participating women responded positively to the mental health support they received, and findings provide important insights into how services might further support their needs.

**Electronic supplementary material:**

The online version of this article (10.1186/s12888-018-1815-7) contains supplementary material, which is available to authorized users.

## Background

Perinatal depression is one of the commonest morbidities of the perinatal period [1]. Left untreated, depression in pregnancy and the post-partum period can have serious and potentially long-standing consequences for women, their children and families [1, 2]. The burden of perinatal depression is significantly higher in many low- and middle-income countries (LMIC), where exposure to chronic adversity and difficult living conditions is greater and access to adequate mental health services is more limited [3, 4]. Migrant populations are at particularly high risk of perinatal depression due to an array of potential stressors experienced throughout the migration trajectory [5, 6]. Global estimates suggest there are currently over 1 billion migrants worldwide, the majority of whom are labour migrants who move primarily in search of employment opportunities [7, 8]. Despite the majority of global migration occurring within LMIC regions, evidence around the perinatal mental health of migrant women has focused largely on high-income destination settings, and research involving women who have resettled from one low-income setting to another is severely lacking [5, 7, 8].

To date, studies around migrant perinatal mental health have focused on quantifying the prevalence and identifying risk factors. Systematic reviews report pooled estimates of perinatal depression prevalence between 19% [9] and 36.5% [5] among resettled migrant women, though these estimates mask considerable variation between sites. Risk factors include socio-economic disadvantage, low levels of social support and experiences of conflict, exploitation and interpersonal violence [5, 10]. Many of the factors associated with perinatal depression also appear to be context-specific and vary according to culture and setting. For instance, traditional birthing practices such as postpartum confinement and a cultural preference for male infants are associated with postnatal depression in some cultures [11]. Less research has is available which explores in greater depth the lived experiences of migrant women with perinatal depression, particularly among those living in LMIC settings. Given the significant populations involved and the potentially wide-reaching consequences of the condition, there is a need for qualitative work to elicit a greater understanding of the views of affected women in resource-constrained settings. This qualitative study addresses the significant evidence gap by exploring experiences of perinatal depression among refugee and labour migrant women living along the Thai-Myanmar border.

## Methods

This qualitative in-depth exploration complements and provides important contextual information for a larger prospective cohort study of perinatal depression, details of which are described elsewhere [12]. In brief, a cohort of 568 refugee and labour migrant women on the Thai-Myanmar border were recruited in the first trimester of pregnancy and followed up until 12 months post-partum. Women’s depression status was determined using the *Structured Clinical Interview for the Diagnosis of DSM-IV Disorders* (SCID) [13]. Participating women who experienced major depression during any stage of their pregnancy were eligible for inclusion in this qualitative study.

### Setting

This research was conducted in Tak Province, located on the Thai side of the Thai-Myanmar border. Decades of civil conflict in Myanmar have led to large-scale displacement of individuals from Myanmar into Thailand and other neighbouring countries. There are currently an estimated 200,000 labour migrants and an additional 145,000 refugees living along the Thai-Myanmar border [14, 15]. In Tak Province, displaced populations are predominantly of Karen or Burman ethnicity with a small minority of Muslim groups. In this setting, refugees live in established refugee camps on the Thai side of the border where they benefit from housing, food rations and access to health and education services provided by non-governmental and community-based organisations. Labour migrants constitute a separate group who move from Myanmar to rural villages along both sides of the Thai-Myanmar border primarily in search of work in agricultural, service or manufacturing industries. The Shoklo Malaria Research Unit (SMRU) has provided maternity services to displaced communities in this region since 1986. The current study was based at two SMRU antenatal clinics: the first located in Maela refugee camp, the largest camp along the border with an estimated population of 38,000 [16], and the second located in Wang Pha and serving the local rural labour migrant community.

### Participants and sampling

Participants were pregnant and post-partum refugee and labour migrant women attending SMRU clinics. The sampling frame consisted of any woman who had participated in the larger cohort study described above and who had been diagnosed with major depressive disorder at any stage during her pregnancy. Eligible women were contacted either by telephone or in person during routine antenatal visits. Efforts were made to contact all eligible women. In an attempt to achieve maximum sampling variation, women from under-represented ethnic, religious and language backgrounds were approached first. Verbal and written descriptions of what the in-depth interview would entail were provided. We explained that participation was entirely voluntary and that participation or non-participation would bear no effect on any aspect of women’s care. Women who agreed to take part provided written consent and an interview time and date was arranged. Interviews were conducted at SMRU clinics and whenever possible occurred immediately following women’s routine antenatal or postnatal clinic appointments in order to avoid incurring additional travel to and from the clinic. At the end of the interview, participants were offered a small gift (baby clothes and soaps) of an approximate value of GBP £2 as a token of appreciation for their time.

### In-depth interviews

In-depth interviews were conducted using an interview guide which covered topics of interest including current and past life experiences, social support and the impact of depression upon social and occupational functioning (Additional file 1). The interview guide was intended to serve as a prompt rather than act as an exhaustive list, and women were encouraged to discuss any other issues they perceived to be relevant to their experiences of depression. Information on participants’ age, ethnicity, religion, educational background and migrant status were also collected.

Interviews were conducted by two researchers: an English-speaking physician (GF) and a social scientist fluent in Sgaw Karen, Poe Karen, Burmese and English and highly experienced in interviewing in this setting (SN). Interviews were conducted in private rooms adjacent to the antenatal waiting areas in Sgaw Karen or Burmese, according to each woman’s preference. In order to put women at ease, interviews began with general conversation around topics such as where the participant came from, their childhood, their journey to the clinic that day and what they enjoyed doing in their free time. Once a good rapport had been established, the focus moved towards women’s experiences of perinatal depression. Participants spoke Burmese or Sgaw Karen, and none spoke English. Questions were asked first in English by GF. The questions were interpeted by by SN into Sgaw Karen or Burmese. Participants’ responses were interpreted by SN back into English. As each interview progressed, follow-on questions were asked either in English by GF or directly by SN in the interview language, such that discussions took on the format of three-way conversations rather than two-way questioning-and-answering via an interpreter.

Interviews were audio-recorded using a digital voice recorder. Immediately following the interview, any impressions regarding participants’ affect, demeanour and non-verbal communication were noted. Interviews were transcribed directly into English by an experienced transcriber and interpreter. Due to a lack of resources it was not possible to use a two-step process of verbatim transcriptions followed by formal translation of the transcript into English. In order to ensure accuracy, a random selection of transcript excerpts was compared with the original audio recordings. Any ambiguous or unclear language was discussed and clarified. Final versions of the transcripts were imported into *NVivo 11* for analysis [17].

### Analysis

A thematic analysis of the data was conducted by one author (GF) using the six consecutive phases described by Braun and Clarke (2006) [18]. First, transcripts were read and re-read until thorough familiarity with each participant’s account was achieved. Then, features of note emerging from the data were coded and collated into potential themes and sub-themes. Themes were reviewed and refined until clearly defined themes were established that worked in relation to each of the coded extracts as well as to the entire data set. In order to maximise reliability of the coding and enhance the consistency and quality of data interpretation, a sample of three full interview transcripts was coded independently by a second author (EP) [19]. Themes identified by the two authors were compared and ways in which subthemes might be grouped together were discussed.

The importance of transparency in qualitative research – including acknowledging researchers’ active role in identifying themes and selecting those considered to be of interest – has been highlighted by Braun and Clarke (2006). For our thematic analysis, we adopted an essentialist (or ‘realist’) approach whereby we report the experiences and lived realities of participants [18]. We do not examine the ways in which such experiences may or may not be the result of discourses operating within the local context [18]. It is also important to acknowledge the potential for researchers to be subject to unconscious biases which may influence data collection and analysis, including how meanings are ascribed to findings. In this study, the interviewers (GF and SN) and data analysts (GF and EP) all have previous experience of working with marginalised populations on the Thai-Myanmar border. One of the interviewers (SN) is of Karen ethnicity and was able to confer a high level of cultural sensitivity to interviews. All three interviewers have experienced life as ‘outsiders’; along with their status as mothers they may have held sympathetic leanings towards this population of pregnant migrant women. Nevertheless, detachment and objectivity was maintained as far as possible as a result of none of the researchers being fully immersed within the local culture.

Concurrently with the coding of data, the ‘one sheet of paper’ (OSOP) method was used. The OSOP method involves reading through each section of qualitative data and noting the different issues raised within coded extracts onto one large sheet of paper. This provides a visual summary of all the issues within a particular code, which might include both anticipated and other, ‘emerging’ issues [20]. In our analysis, separate sheets of paper were used for each theme identified in women’s narratives. All statements relating to a particular theme were noted, allowing similarities and discrepancies between participants to be highlighted and any examples of unexpected and deviant narratives to be identified. The identification of deviant narratives in particular serves as a means to ensuring that a critical stance is maintained by researchers throughout the analysis [20]. Quotations from women’s interview transcripts were used to highlight themes in the presentation of results. Often in qualitative research, such quotes are contextualised by providing a number of key socio-demographic characteristics of the participant. Because of the small number of women interviewed for this study and the sensitive nature of discussions, only a numeric identifier is provided alongside each quote in order to avoid compromising anonymity.

### Ethical approval

Ethics approval for this study was granted by the University of Oxford Tropical Research Ethics Committee (OxTREC 28–15), Mahidol University Faculty of Tropical Medicine Ethics Committee (TMEC 15–045) and the Tak Border Community Advisory Board (T-CAB 6/2/2015).

## Results

Eleven women with severe perinatal depression participated in in-depth interviews. This final sample represented a convenience sample of women whom we were able to contact and who agreed and were able to attend for an interview. Of a total of twenty eligible women who had been diagnosed with severe perinatal depression within the larger cohort study, thirteen could be contacted. Of these, one declined to be interviewed because she had moved far away from the clinic, and one agreed to participate but did not attend at the arranged interview time. The remaining eleven completed in-depth interviews. Contacting women was considerably easier at Maela refugee camp, as camp residents live within established confines and generally have easier access to the clinic. Labour migrant women – a geographically more mobile group – were comparatively more difficult to contact. As a result, refugee women were over-represented in the final sample. Interviews were carried out between May and July 2016 and ranged in length from 45 min to one and a half hours, with an average duration of just over one hour.

Socio-demographic characteristics of participating women are summarised in Table 1. The majority of participating women were of refugee (*n* = 9), Sgaw Karen (*n* = 8) and Buddhist (*n* = 6) background and most were interviewed in their third trimester of pregnancy (n = 6). The majority of women had been displaced from their homes over six years ago, and only two women had been displaced for a period of less than five years.

**Table 1:** Characteristics of participants of qualitative study component (*n* = 11)

Characteristics	No. of participants
Age	
20–24 years	2
25–29 years	3
30–34 years	1
35–39 years	3
40–44 years	2
Migration category	
Refugee	9
Labour migrant	2
Ethnicity	
Sgaw Karen	8
Burman	2
Burman Muslim	1
Religion	
Buddhist	6
Christian	4
Muslim	1
Years of education received	
Less than 3 years	4
3 to 6 years	3
7 to 10 years	4
Parity	
1	2
2–4	5
≥5	4
Stage of pregnancy	
2nd trimester	2
3rd trimester	6
Post-partum	3
Duration since migration	
5 years or less	2
6 to 10 years	4
Over 10 years	5

A number of themes and subthemes emerged from in-depth interviews with women. The four main themes were: relationships with partners (encompassing the subthemes of quality of relationships; alcohol use; and interpersonal violence); ‘difficult lives’ (encompassing the subthemes of past trauma; present challenges; and uncertain futures); coping strategies; and impressions of mental health care.

### Relationships with partners

One of the key themes to emerge from in-depth interviews was women’s relationships with their partners. When women described relationships with partners, three main subthemes emerged: the quality or nature of their relationships with partners, the use of alcohol among partners and experience of interpersonal violence within their relationships.

Among this group of severely depressed women, a small minority described positive partnership relationships. These women reported that their partners made them feel *“looked after”* and *“cared for”*.

*“We were in love with each other… Nobody in our family supported us to go to school. We both went to work as daily labourers and supported each other.”* (Participant 2)

The majority of women reported being unhappy in their relationships. These women described a perceived lack of attention, empathy and understanding from their partners, resulting in feelings of sadness and frustration. Strained relationships led to feelings of loneliness and isolation, and a sense of having *“nobody to talk to”*.

*“I felt really sad, so I told my husband about it. But you know what he said? He asked me, ‘What happened?’ Just this one word he asked. I told him how I feel, but… he said, “You [feel] like this because you are not supporting your family.” He never comforts me, only blames [me].”* (Participant 10)

*“I have no one to talk my worries out of my chest… There is no one who will listen to me patiently. There isn’t anyone who will come to me and ask what I am going through. Every worry I feel is kept only inside my chest.”* (Participant 5)

Many women blamed negative attributes and behaviours of their husbands for their poor relationships. Men were commonly described as lacking in drive and motivation, and as being unsupportive of women in their roles of caring and household responsibilities, leading to a sense of frustration among women.

*“He doesn’t like to work. If he were a hard worker, there [would] be an income. If you just go and find a job and get hired, it’s easy to get an income… But he is too lazy.”* (Participant 9)

*“Once he finishes with his work, he would go and play volleyball and he comes home at 6pm. I have to wait for him for dinner and I would get upset at him… When I get home and there is unfinished housework or an empty water bucket, I have to finish doing the work and fill the water buckets myself.”* (Participant 2)

Alcohol use in men was common and invariably associated with negative impacts upon relationships with partners. Frequently, the immediate effects of drunkenness were regarded by women as embarrassing and as bringing shame upon their family.

*“Each time [my husband] drinks I just want to die. He becomes very loud and it is humiliating.”* (Participant 2)

Longer-term implications of alcohol use, including the wasting of household income on alcohol, brought about feelings of stress and *“overthinking”* among women.

*“I am depressed when he drinks alcohol and then I [start] overthinking. I would cry every time he drank alcohol. I am already preoccupied enough taking care of just one child.”* (Participant 2)

*“He is always getting drunk and looking for trouble… I am very stressed because of him.”* (Participant 3)

Women described the negative influence of their husbands’ peers on alcohol consumption. Many women felt that their husbands tended to drink more when they were out socialising with friends who might initially offer free drinks, encourage heavy drinking, and tease those men who did not drink.

*“He sometimes uses drugs and alcohol because his friends make him.”*(Participant 5)

*“His friends made him drink… [They] told him he is afraid of his wife. So, he drank the alcohol to show them that he is not afraid.”* (Participant 11)

Poor relationships with partners were also characterised by interpersonal violence, which was reported by almost all the women interviewed and almost always occurred against a background of partners’ alcohol use. Women described instances of verbal as well as physical and, to a lesser extent, sexual, abuse. Verbal abuse included *“hurtful words”* and *“accusations”*:

*“[My husband] says that I am not a good mother and I am hurt by that. When he says that, it hurts me so much that I would cry.”* (Participant 2)

*“He threatens to cut my throat... He also shouts at me with anger and some abusive words and I feel very frightened and shocked because I just delivered the baby and [the baby] is very young. He has no sense of understanding that at this time women need to keep warm and avoid shocks.”* (Participant 3)

Physical abuse included being kicked, hit, punched and strangled. Such attacks were commonly severe enough to cause serious injury and require hospitalisation.

*“He physically abused me twice. The first time, he did not know I was pregnant. He hit my head with a bamboo stick and he kicked me. The [second] time he already knew that I was pregnant… He was coming home after going to get the wood and I forgot to cook and he got angry and kicked me which left me unconscious.”* (Participant 7)

*“He beat me up and squeezed my neck. That time, my eldest daughter ran […] to call for help. After the fighting ended, I was not feeling well for four days so I went to the clinic.”* (Participant 6)

Violence by men was at times directed at their children. When this occurred, women felt a deep sense of shame, helplessness and guilt at being unable to protect their children.

*“Sometimes I feel worthless… When my husband shouts at [the children] I think it’s all my fault… [Because] I am not good, my children are being scolded and beaten by their step-father. I don’t blame my children; I blame myself.”* (Participant 5)

Sexual violence was reported less frequently. One woman recounted how she had felt pressured into marrying her husband after he kissed her in public – an act deemed to be appropriate only among married couples in this cultural context. This woman described feeling vulnerable as well as *“angry”* at the situation she was placed in, which had left her with no choice other than to marry.

*“I was 18 or 19 years old. He was kissing and hugging me in front of people and I was afraid that people will gossip about me so I had to marry him.”* (Participant 7)

Though less common than male-perpetrated violence, women occasionally acted violently towards their partners. A number of women had spoken out against their partners when they could no longer refrain from doing so.

*“I feel so irritated. Right now, I can’t just be silent, you know … I have to speak out to him! I felt great and empty in my chest when I called him a dog!”* (Participant 6)

*“When I get upset and stressed, I have to speak about it, otherwise I would feel like it was burning inside. I would feel better afterwards.”* (Participant 2)

Women often laughed while recollecting the occasions when this had occurred, conveying a sense of embarrassment but also of achievement for having stood up for themselves. The act of releasing pent-up anger and frustrations brought a sense of relief and empowerment. Some women had become physically violent towards their partners. Most commonly this was in direct self-defence against husbands’ abuse, but at other times it was in reaction to behaviours such as repeated drunkenness which women felt were irritating and unacceptable.

*“When [my husband] came back, I told him not to come upstairs… I [made] him stay downstairs until he wasn’t drunk anymore… But he came upstairs. I couldn’t bear his smell and got angry when he didn’t listen. I threw things…at him. If I had not been held back by other people, my husband would have been hit pretty hard… I [insulted] him by calling him: ‘Dog! You animal!’… People say that my husband is going to die from the things I throw at him.”* (Participant 6)

*“My husband and I had an argument one day. I said, ‘I wish I could kill you and take your heart out,’ and I punched him in the eye because I [was] very angry… Since then, he is very terrified of me and apologized [to] me. He said to me: ‘My wife, please don’t kill me!’”* (Participant 7)

“Difficult life”

All women referred in one way or another to their lives being *“hard”*, *“difficult”* or *“tough”*. The concept of a ‘difficult life’ encompassed three subthemes: past traumas, the multiple challenges of daily life and uncertain futures.

Women’s pasts were fraught with hardship:

*“If I think about [my childhood] I don’t even want to live… There were so many hardships.”* (Participant 1)

One woman described growing up with an abusive step-father. When he touched her inappropriately, she found herself unable to find support or share her distress.

*“He touched me inappropriately… I do not like to sleep with blanket on and whenever he came over to put the blanket on me he would touch my breasts and my lower body part and [when] I opened my eyes he walked away. I was upset with what happened. My mother did not let me report [what my step-father did] to the soldiers, so I did not, so that she would not lose face.”* (Participant 2)

Death among parents and siblings was common, and many women’s childhoods were shaped by the absence of a mother or father. As young children, these women had taken on adult responsibilities such as looking after younger siblings, carrying out household chores and being sent as far as Bangkok to earn an income from a young age.

*“After our mother passed away, I had to take on my mother’s [role] of taking care of my younger sister and my father.”* (Participant 5)

“*I was sent [to Bangkok] as a house-maid. I was twelve years old at that time*.” (Participant 10)

*“I went to Bangkok with other people. I started working there at nine years old. I did the cleaning and ironing… I was there for one year.”* (Participant 3)

Many women had not attended school, or attended only intermittently or for brief periods. Often, family finances were insufficient to cover the costs of tuition, uniforms and textbooks. In addition to saving the family money, not enrolling a child in school meant the child was available to help with domestic chores, looking after other family members or potentially earn an additional income for the household.

*“I was only able to go to school for the first grade post-kindergarten. My family had to struggle very hard. They could not afford for my school fees easily. I enjoyed school. I [tried] my best when I was in school.”* (Participant 5)

Conflict and war in Myanmar and the resulting displacement to the Thai border represented, for many, an additional past trauma. The constant threat of violence from Myanmar government army soldiers as well as armed ethnic minority groups was a significant stressor. Women had experienced and witnessed multiple forms of violence, including forced labour, portering and minesweeping, land confiscation and killings.

*“In my area, the Burmese soldiers came and collected people as their porters. As they were going in and out of our village day and night, we did not dare to go work so that our living conditions started to become unstable and poor.”* (Participant 8)

Many families had been forced to flee their homes and villages, often very suddenly, taking nothing but *“the one set of clothing on my body”*. Women remembered their arduous journeys to the Thai border in detail even when these events had occurred decades earlier.

*“There was an attack in which we had to flee from the Myanmar soldiers. There was an exchange of fire and I had to flee with my two younger siblings. My mother was carrying another sibling and my brother was asked to carry clothes. I carried the cooking pot and some kitchen accessories and some rice… There were many other items which we could not carry.”* (Participant 2)

*“We left because of the civil war. We had to stay in the bunker… Fighting broke out and my mother was three months pregnant at that time. We crossed the border on foot.”* (Participant 3)

*“It probably took about 4-5 days on the way because we had the financial difficultly and my father was not well on our journey. After our mother passed away, I had to take the substitution of my mother’s position for taking care of my younger sister and my father. So, we hitchhiked and walked and begged for food.”* (Participant 5)

#### Present challenges

Present life challenges were often the result of long-standing poverty and income insecurity. Women worried about not having enough food to feed the family and many were unable to afford school fees.

*“Even with our income and monthly ration, we don’t have enough for my family. I have to buy one sack of rice once every two months and two sacks of charcoal every month.”* (Participant 4)

One woman described feeling sad because she could not afford to buy her children small treats. It was common for families to take out loans from friends or informal moneylenders in attempts to make ends meet. Often these loans would set off a downward spiral of mounting debts that women were unable to repay. Many women felt that the responsibility of taking care of the family rested solely upon them, and this created resentment towards partners as well as acting as a constant psychological stressor.

*“I am the only person providing for the family. I also have to provide for my parents because there is no one else. When I can, I send money to my parents but when I do not earn enough I could not send them any.”* (Participant 4)

*“I work every day… With a bit of money that I…earn at work I have to pay for the babysitter. My father, when he comes around, he asks me for some money to buy alcohol. If I refuse, he starts to become violent and destroys what he sees around. I get some money from my husband but I feel mistrusted by him at the same time. They are putting me under many pressures.”* (Participant 3)

Family separation characterised many women’s lives. It was common for women to have lost contact with close family members including siblings, parents, former partners and even their own children who had remained behind in Myanmar, left in search of work opportunities or resettled to third countries.

*“I look after three of my nieces and nephews. Their father was left behind and their mother is having difficulties getting a job so we have to help look after them.”* (Participant 2)

*“The eldest daughter lives with me. The other four children are with my previous husband. He does not care where they go or what happens to them. He lets them go to school but he dislikes them visiting me, so he beats them and makes them stay with his sister. I am uncertain how well they look after my children.”* (Participant 8)

#### Uncertain futures

Among this group of women, most of whom were of refugees (rather than labour migrants), the majority hoped to one day resettle to ‘third countries’ – a term used to describe high-income destination countries including the United Kingdom, Australia and the USA. However, none of the women had a clear sense of how likely it was for their applications for resettlement to be successful. Those who had not been registered with the refugee camp’s UNHCR resettlement scheme expressed doubt as to whether they would ever be allowed to move to another country. But even among those on the resettlement register there was great uncertainty. Some women had been waiting for several years, with no indication of if or when they might move. Others had complicated scenarios whereby only some family members were registered for resettlement, creating difficulties for women faced with the choice of moving to a new country alone or remaining in the camp with their families.

*“I wish to resettle if I could. I want to go to another country because all my parents and siblings are there. Here, I am not healthy. I am overwhelmed and worried about the future. I wish to go to my parents so that they can also help take care of their grandchildren.”* (Participant 5)

Repatriation to Myanmar was not seen as an attractive option due to ongoing conflict and difficult living situations in Myanmar:

*“I haven’t thought about going back to Burma and I don’t want to. I will get some land here if I live here. Wars are still breaking out in my old village and we [would] have no place to go.”* (Participant 6)

*“I don’t want to go back [to Karen State]. I do not like living there. I would have to work hard if I moved back. It is better to live here.”* (Participant 7)

The overarching issue of a “difficult life” encompassing the multiple challenges pervading every aspect of women’s lives is summed up by the following two quotes:

*“My life situation is [similar to] that of the fruit of the Devil’s Trumpet: if you swallow it, it will sting you; if you smell it, it will make you dizzy; and if you eat it, it will make you mad. My life is like this: I have no direction I can go, nowhere to run away or escape this trouble. Sometimes I think I want to choose the path of suicide.”* (Participant 5)^1^

*“Everyone has their limit and if the burden is too much then you would no longer be able to tolerate it. There’s no need to say it: I have so much burden in this life… Even though I have made a lot of good merit and deeds I have never been lucky. I am carrying this very heavy burden. Sometimes it feels like I cannot take it anymore.”* (Participant 7)

### Coping mechanisms

Women resorted to a variety of mechanisms to help them cope with symptoms of depression. A number of women sought emotional support from friends, neighbours and work colleagues. One woman received support from her neighbour who could relate to and empathise with her situation.

*“She is like a sister and a mother [to me]. Like me, she is also feeling unwell with sadness. When I talk about my problems, she feels sad for me, and I feel the same when she tells me her problems. There is nothing I can do. Besides her, there is no one I could talk to.”* (Participant 5)

Other women turned to hobbies including weaving, embroidery and gardening as a means of momentary escape. For some, provided a welcome distraction.

*“I seem to forget about my worries when I concentrate on work that I enjoy.”* (Participant 5)

*“I forget [my symptoms]…while I am in work. Honestly speaking, I didn’t want to come home from there. I wanted to live but there wasn’t a room for me. Sometimes, I called my co-worker to ask whether or not I could sleep there because I didn’t want to go back.”* (Participant 10)

For a small number of women, religious activities such as reading the Bible and visiting the church, temple or mosque were perceived as helpful ways of managing symptoms of depression, though more commonly these activities were mentioned in passing rather than being regarded explicitly as coping strategies.

*“When I am home, I am not very happy but when I go to church and hear about God’s words I feel happy. When I come home, the devil comes to my heart all the time. Whenever my friend invites me to join her to church, I would go but if I refused the invitation and did not go, the devil would come then.”* (Participant 7)

A couple of women had consulted traditional healers with regards to their symptoms of depression. These women felt that the treatment had helped them to feel more positive and balanced, and given them a more positive perspective on their lives.

*“A traditional healer made this necklace for me to protect further bad deeds and evil spirits causing me [to be] unwell. The necklace holds a small piece of scroll that is written in traditional magic words to cast evils away and protect me. While I am wearing this necklace, I mustn’t go to places that are unclean and I mustn’t eat anything that is unclean.”* (Participant 5)

*“When I felt sad I went to the clinic and had an injection to help with stress. I always pray to lose this symptom of feeling sad when I meet with a good healer because I have been feeling really bad.”* (Participant 11)

### Impressions of mental health care

Counselling offered to women was generally well-received. For many women, counselling provided a rare opportunity to talk about their feelings Women felt it particularly beneficial to speak to the same counsellor regularly over several months, which allowed them to build up rapport and trust. As well as the counselling sessions in themselves, spontaneous conversations that arose with other women while they were waiting for counselling were also helpful.

*“This morning, some women were talking about their problems. Sometimes you see that some of them are going through the same problems as [me]. The person I talked to this morning, her husband [gets himself into] trouble…and my husband also steals money… I realise that I am not alone going through the burden of these difficulties, but that there are more people like me.”* (Participant 6)

Of the eleven women interviewed, all had been offered anti-depressant medication for their severe depression and seven chose to take the medication. Women who took medication reported mixed experiences. Some reported positive experiences: these women found the treatment helpful, describing that it contributed towards an improvement in their symptoms and that they felt “*more active”* and less worried following treatment.

*“Since taking the medicine I can sleep well and eat well. I now think less about my worries. I suddenly feel good. I have a good appetite and I can eat well. I also do not worry about my children like before. The thoughts do not stick in my mind for too long.”* (Participant 8)

*“When I take the medicine, I feel better. I feel less tired. It could be other things that have helped me feel better, but personally I think it’s because of the medicine. I can laugh…now. Before I took the medicine, I couldn’t.”* (Participant 11)

Others experienced side effects and discontinued taking the medication. Reported side effects included feeling *“uncomfortable”*, *“woozy”* and *“numb”*. One participant felt better when she took the medicine, “*but when I stop taking it the thinking creeps back in”*.

## Discussion

This qualitative study was conducted in order to gain a deeper understanding of refugee and labour migrant women’s experiences of severe perinatal depression on the Thai-Myanmar border. Participating women provided extensive insight into the many difficult aspects of their lives that they perceived as contributing to their depression status and the coping mechanisms they adopted in order to help them cope with the condition. A complex constellation of overlapping and inter-connected issues shaping women’s experiences arose from women’s narratives. The concept of a “difficult life” encompassed a variety of on-going but also past social, economic and interpersonal challenges as well as uncertainty regarding the future. Women’s lives were to a large extent shaped by past events including difficult childhoods, family separation, conflict and dangerous journeys to the Thai border. As adults, these difficult life circumstances persisted in the form of economic hardship, insecurity, poor support from partners and a lack of certainty around their own futures.

Another central tenet of these women’s experiences was difficult relationships with partners, linked closely with partner’s alcohol use and interpersonal violence. Strained relationships were common, and men’s alcohol consumption formed one of the main frustrations women had with their partners. Alcohol use was regarded as leading to inappropriate behaviour in men, ranging from being unsupportive emotionally and financially to bringing shame on the family through drunken behaviour in public, all with negative impacts upon women’s lives. Women were exposed to interpersonal violence in the form of verbal and physical abuse and, to a lesser extent, sexual abuse, and violence was invariably associated with and aggravated by men’s alcohol use. The relatively low frequency with which women reported sexual violence is of interest given that the perinatal period has been highlighted as a time of high risk for violence against women [21]. It is possible that women under-disclosed episodes of sexual violence as a result of stigma around this issue or a lack of clarity around definitions of what constitutes sexual abuse [22]. Coping mechanisms included talking to friends and family as well as work, hobbies, religious activities and traditional healing.

The importance of psychosocial factors parallels findings from other settings. Previous research has highlighted the significant contribution of living conditions, interpersonal violence and social support towards perinatal depression among both migrant and non-migrant populations [1, 5, 11, 23]. The inter-association between alcohol use, interpersonal violence and partner relationships has also been reported in other resource-constrained settings, including elsewhere along the Thai-Myanmar border [24–27]. In a previous survey of pregnant women in Maela camp, almost a quarter of women reported high levels of alcohol use by their partners [27]. Similarly, in a different refugee camp on the Thai-Myanmar border, alcohol use and fighting within married couples emerged as predominant stressors [26]. Paralleling the findings of the current study, Meyer et al.’s (2013) respondents attributed relationship discord to multiple, inter-related problems including a lack of income, having nothing to eat, alcohol use and frustrations with general conditions within the refugee camp.

The notion of a ‘difficult life’ suggests that women in this study represent a particularly vulnerable group as a result of exposure to multiple co-existing and mutually-reinforcing psychosocial challenges. Other studies have reported similar findings. For example, Stewart et al. (2014) found that in Malawi, a lack of support from partners was perceived by pregnant women as a generalised insufficiency of support in their lives, which in turn was associated both with depression and interpersonal violence [28]. Similarly, Fernando et al. (2010), working with conflict-affected Sri Lankan youth, ascribed depression to a “host of enduring stressful conditions of daily life” [29].

Women found therapy in the form of counselling helpful, especially when rapport could be established with the same counsellor over a period of time. The mixed reports with regards to anti-depressant medication suggests more research is required to explore effective and culturally-accepted forms of support within this population. Evidence from other low-income settings has shown the effectiveness of psychosocial interventions delivered by non-specialists and peer supporters [30]. Future work might consider testing such interventions on the Thai-Myanmar border.

It was notable that pregnancy status per se did not feature prominently in women’s narratives when, arguably, it might be an important determinant of a woman’s emotional state. In this particular context, this could manifest in either a positive manner – for instance, pregnancy and motherhood providing a reason for hope in an otherwise difficult life situation – or in a negative manner – for instance by highlighting and bringing to the forefront of women’s minds the on-going challenges they face with little prospect of improvement in the next generation. Within Karen culture, childbearing is considered a fundamental aspect of womanhood: marriages are ‘blessed’ with having children, and the idea of attributing symptoms of depression on pregnancy may feel inappropriate to women in this context. The lack of explicit mention of pregnancy and motherhood also raises questions around the extent to which experiences of depression among this group of women are in fact tied to pregnancy status, or whether the condition would have occurred regardless. A small number of studies exploring the mental health of women refugees have reported vulnerabilities that are common to all women, regardless of pregnancy status [31, 32]. However, no studies to our knowledge have directly assessed the specific contribution of pregnancy or post-partum status as a contributing factor to mental disorder among migrant or refugee women.

Evidence is also lacking around the experiences of non-migrant Karen and Burmese women’s experiences of perinatal mental disorders. Without an in-depth understanding of depression within these cultural groups in their places of origin, it is difficult to attribute the experiences of the women included in this study to their migration histories. Future research should consider the inclusion of non-perinatal women within this migrant community as well as non-migrant women in communities of origin within Myanmar in order to explore the relative contributions of pregnancy status and migration status, respectively.

### Limitations and strengths

Findings of this qualitative study are constrained by a number of limitations. Firstly, results are based on a limited number of interviews with a selected group of severely depressed women, and findings may therefore not be generalisable to other women with perinatal depression in this setting. In particular, women interviewed did not reflect the diversity within the migrant community as a whole. Despite attempts to include women from a diverse range of backgrounds, refugee, Sgaw Karen and Buddhist women were over-represented. The small sample size also meant we were unable to provide socio-demographic details of participants alongside their quotes, making it difficult to interpret whether factors such as migration status or cultural background influenced the experiences of these women. A larger and more diverse sample may have enabled differences in experiences of perinatal depression according to factors such as ethnicity, religious background and migrant category to be discerned. However, the small overall number of severely depressed women from which our participants were drawn (*n* = 20) along with the difficulty in contacting many of the women in this highly mobile and hard-to-reach population group limited the ability to select women from a range of backgrounds.

In order to participate, women had to be contactable, willing to participate and available to attend the clinic for interview, introducing the possibility of recruitment and self-selection bias. Importantly, women whom it was not possible to contact may have been the most vulnerable or the most severely depressed. The limited number of participants also meant that data saturation was not achieved, and important factors contributing to experiences of depression may therefore have been missed.

A further limitation relates to language. Interviews were conducted in a combination of English, Karen and Burmese. Compared with English, both Karen and Burmese languages have a limited vocabulary for describing emotional states. It is possible that despite best efforts to minimise losses in translation, inconsistencies across languages or misinterpretation of descriptions of depressive experiences occurred. Furthermore, analyses were conducted in English rather than in the language the participants used, which may have led to subthemes being missed or misplaced.

Finally, though this was beyond the scope of the current study, findings could have been strengthened through the inclusion of women’s partners in order to gain the perspective of the men living alongside these women. Further insights could also be gained by including non-depressed women, many of whom might be exposed to the same stressors. These ‘control’ women may help our understanding of factors contributing to resilience against mental disorders in this setting.

Despite these limitations, this study has a number of strengths. To our knowledge, this qualitative study represents the first in-depth exploration of the experiences of perinatal depression among refugee and labour migrant women in a low-income setting. A number of ethnographic studies of perinatal depression among migrant women exist but all to date have been conducted in high-income settings [33]. A further strength was that one of the interviewers was a trusted member of the migrant community with valuable first-hand understanding of the cultural context, which allowed a strong and trusting rapport with participating women to be established. Finally, analysis was conducted using a rigorous methodological approach. An inter-rater reliability exercise was conducted in which a second author (EP) independently coded three transcripts in order to maximise reliability, while the grouping of sub-themes into themes and the inter-relationship of themes with each other were conducted in consultation and collaboration with the wider team of authors. An explicit acknowledgment of the analytical approach adopted and the inclusion of deviant examples through the OSOP method further helped to ensure that a critical stance was maintained.

## Conclusions

This qualitative study explored themes emerging from the narratives of refugee and labour migrant women living with severe perinatal depression on the Thai-Myanmar border. Women provided considerable insight into their lived experiences of depression and linked their symptoms to a wide range of social factors. Participants faced a wide range of chronic stressors at the individual, household and community levels that are likely to have both short- and long-term effects on their mental well-being and day-to-day functioning. Themes included challenging life situations, difficult partnership relationships marked by alcohol use and interpersonal violence and mechanisms for coping with perinatal depression. Severely depressed women responded positively to mental health counselling support, and these interviews have provided important insights into how services might further support these women’s needs.

## Additional files

Additional file 1: Interview Guide: interview guide used during qualitative interviews. (DOCX 15 kb)

Additional file 2: Dataset Application Form: form for requests to access data used for this study. (DOCX 13 kb)

## Footnotes

1 Devil’s Trumpet (also known as datura or jimsonweed) is a highly toxic genus plant belonging to the vespertine flowering plants species.
