# Supplementary material for: Living with severe perinatal depression: a qualitative study of the experiences of labour migrant and refugee women on the Thai-Myanmar border
Source: BMC Psychiatry. 2018 Jul 16;18:229. doi: 10.1186/s12888-018-1815-7 (PMC6048862; doi:10.1186/s12888-018-1815-7)
Supplement: Supplementary file 1 — Interview Guide: interview guide used during qualitative interviews. (DOCX 15 kb) [file 12888_2018_1815_MOESM1_ESM.docx]

**Interview schedule**

The following interview schedule will be used to guide the interview but we encourage participants to discuss any other aspects they feel are relevant to their experiences of depression.

Opening (to be used selectively, depending on conversation flow)

- Tell me a little bit about yourself. Where were you born? What was your childhood like? Why did you leave your home town, and how did you end up here?
- Who lives with you now?
- What are your hobbies?

Question guide (to be used as prompts only; allow participants to speak freely beyond any of these issues)

- How/in what way does low mood affect your everyday life?
- Can you tell me what it’s like for you, living with depression?
- What do you think are the reasons for your depression/the way you are feeling?
- What are the things you cannot do (or find difficult to do) when you experience low mood?
- What is the most difficult aspect of having low mood/depression?
- Is there anything that helps you at times when you are feeling very sad or unable to cope? / When things are difficult, what do you do?
- When things are difficult, do you manage to do your normal work or take care of things? Do you get any help?
- Do you talk to friends/family about how you feel? Do you find it easy to talk to friends/family about depression?
- Do you find it helpful to speak to counsellors/doctors at SMRU? If so, do you prefer talking alone, with your partner, in a group?

Close

- Any questions you would like to ask us?
- Anything you feel we’ve missed/not covered?
- Give thanks
